# Molecular Biology: Telomerase Tells on Lifestyle

**Published:** 2008-12

**Authors:** M. Nathaniel Mead

Being overweight, stressed, and sedentary have all been shown to accelerate the shortening of telomeres, DNA–protein complexes at the end of chromosomes that protect the genetic material and promote chromosomal stability. A new pilot study now finds that certain lifestyle changes may promote telomere integrity and length by significantly boosting telomerase activity in human immune cells.

Chromosomal stability is strongly correlated with longevity. Thus, if telomere length decreases prematurely, so also does life span. Shortened telomeres have been associated with a heightened disease risk (e.g., a 100-fold higher incidence of vascular dementia) and increased mortality rates for cancer and heart disease. Moreover, evidence published in the March–April 2006 issue of *Urologic Oncology* indicates that by initiating chromosomal instability, short dysfunctional telomeres may participate in prostate carcinogenesis. The leading hypothesis is that telomere attrition is influenced by oxidative stress (which can result from excessive body weight and stress as well as exposure to radiation and certain pollutants, such as airborne particles) and by inflammation (which generates and is perpetuated by oxidative stress).

Telomerase, an enzyme that directs the replication of telomeres, adds telomeric repeat sequences to the chromosomal DNA ends, explains principal investigator Elizabeth Blackburn, a molecular biologist at the University of California, San Francisco. “This preserves not only telomere length, but in the case of cells of the immune system, also healthy cell function and long-term immune function.” Nevertheless, she adds, “We do not yet know whether increasing telomerase activity could help reverse the problem of age-related telomere shortening.”

The study, reported online 15 September 2008 ahead of print in *Lancet Oncology*, assessed whether lifestyle changes could increase telomerase activity in peripheral blood mononuclear cells (PBMCs), an infection-fighting component of the immune system. The authors hypothesized that improvements in nutrition, exercise, and stress management—lifestyle factors thought to help prevent cancer and cardiovascular disease—might also bolster telomerase function.

Thirty men with biopsy-diagnosed, low-risk prostate cancer attended a three-day retreat followed by an outpatient phase. The men were asked to make comprehensive lifestyle changes: 1) adhere to a low-fat, primarily vegetarian diet supplemented with soy protein, fish oil, selenium, and vitamins C and E; 2) engage in moderate aerobic activity 30 minutes per day, 6 days a week; 3) practice stress management techniques such as yoga stretching, breathing, and meditation 6 days a week; and 4) attend a 1-hour support group once a week. Adherence was assessed by questionnaire. Changes in telomerase activity were measured at baseline and after 3 months.

Among 24 patients with sufficient PBMCs for longitudinal analysis, two-thirds showed a significant (29–84%) increase in telomerase activity, an indication of telomere restoration. This increase was also associated with significant decreases in low-density lipoprotein cholesterol and in one measure of psychological distress (intrusive thoughts). There were no significant changes in total levels of prostate-specific antigen, the marker for prostate cancer, nor was there evidence of cancer progression in the group.

“It seems remarkable that decreased stress and improved diet might give rise to measurable increases in telomerase activity in such a short time period,” says University of Glasgow biologist Pat Monaghan, who with Mark Haussmann coauthored a report on lifestyle and telomere dynamics in the January 2006 issue of *Trends in Ecology & Evolution*. Robert Sapolsky, a neuroendocrinologist at Stanford University, adds that the study “shows that [an] undesirable telomerase profile can be reversed with the sorts of lifestyle interventions that decrease the risk of various stress-related diseases.”

The potential confounding factors and limitations of the study—the small size and short duration of the study, the complexity of the lifestyle changes, the absence of any data on the effect on telomere length, and the lack of an independent control group—make it impossible to identify causal factors. “Since these lifestyle changes and biological factors all tend to interact and reinforce each other, it’s difficult to determine which matter most,” says coauthor Elissa Epel, an associate professor of psychiatry at the University of California, San Francisco. Moreover, says Monaghan, the study involved a broad age range of men, and it is unknown whether the effects varied with the age of the patient.

Nevertheless, says Epel, the findings point to some provocative possibilities. “If we can improve our health behaviors, we might be able to reverse telomere shortening or at least stabilize the telomeres in our blood,” she says. Parsing out the most critical parts of the intervention is an important direction for future research. For now, Epel says, the take-home message is that what’s good for promoting cardiovascular health also seems to help curb cellular aging. This knowledge, in turn, could help motivate people to change their diet, exercise, and stress management habits for the better.

## Figures and Tables

**Figure:**
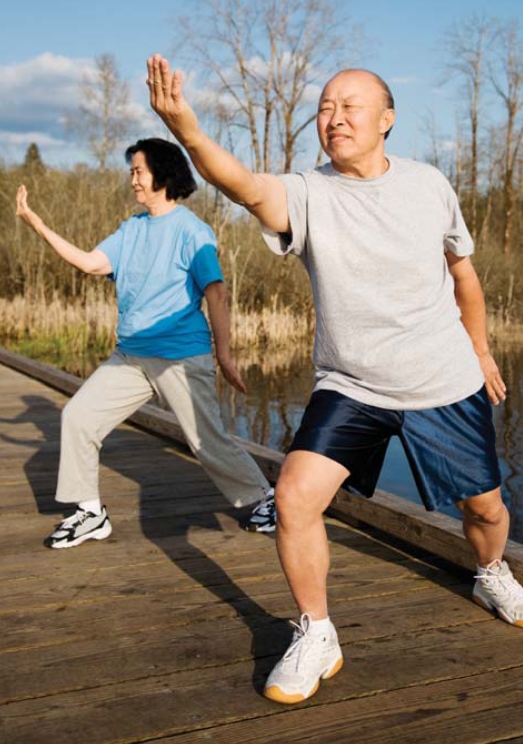
Diet, stress, and exercise are important factors in chromosomal health.

